# Supplementary material for: Parental Self-Efficacy and Child Diet Quality between Ages 2 and 5: The STEPS Study
Source: Nutrients. 2022 Nov 18;14(22):4891. doi: 10.3390/nu14224891 (PMC9698359; doi:10.3390/nu14224891)
Supplement: Supplementary file 1 [file nutrients-14-04891-s001.zip › nutrients-1999643-supplementary.pdf]

Table S1. Characteristics of STEPS study families. Comparison between participants (longitudinal data) and non-participants based on percentage and chi-square test for categorical variables and with means and t-test for continuous variables.

Variable		2 years age point	Non-participants	Longitudinal sample [1]	*p*-value
		n = 580	*n* = 307	*n* = 273	
**Children**					
Sex					0.98
	Boy	312 (54%)	165 (54%)	147 (54%)	
	Girl	268 (46%)	142 (46%)	126 (46%)	
Siblings					0.53
	0–1	484 (83%)	259 (84%)	225 (82%)	
	2 or more	96 (17%)	48 (16%)	48 (18%)	
Diet quality		6.10 ((1.69)	6.04 (1.78)	6.17 (1.59)	0.33
**Mothers**					
Age					0.31
	17–29	229 (40%)	127 (42%)	102 (37%)	
	30–45	350 (60%)	179 (59%)	171 (63%)	
Education					0.20
	Low	182 (32%)	102 (35%)	80 (30%)	
	Advanced	384 (68%)	193 (65%)	191 (70%)	
PSE		85.73 (8.45)	85.85 (8.55)	85.60 (8.35)	0.73
**Fathers**					
Age					0.59
	17–29	157 (27%)	86 (28%)	71 (26%)	
	30–45	423 (73%)	221 (72%)	202 (74%)	
Education					0.84
	Low	269 (48%)	140 (47%)	129 (48%)	
	Advanced	295 (52%)	156 (53%)	139 (52%)	
PSE		82.71 (10.13)	82.30 (10.96)	83.18 (9.10)	0.30
**Families**					
Income					0.77
	<3000 EUR	298 (54%)	156 (54%)	139 (51%)	
	≥3000 EUR	252 (46%)	135 (46%)	133 (49%)	
Education					0.38
	Low	133 (23%)	74 (25%)	59 (21%)	
	Advanced	439 (77%)	225 (75%)	214 (79%)	
PSE		84.22 (7.46)	84.07 (7.90)	84.39 (6.94)	0.61

[1] Longitudinal sample = 2 years ja 5 years age points. STEPS, Steps to Healthy Development; PSE, parental self-efficacy.

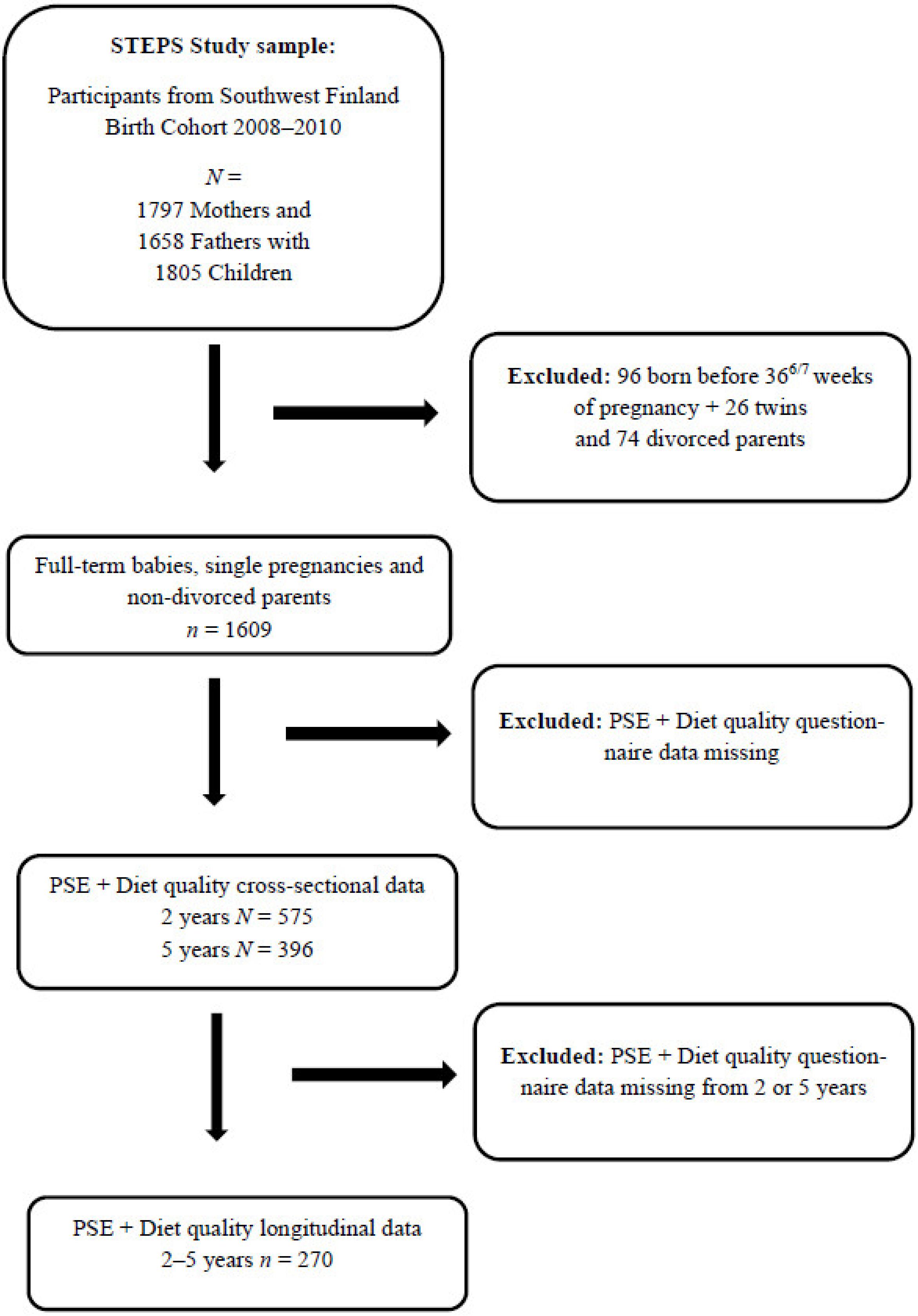

Figure S1. Flowchart of the study. STEPS, Steps to Healthy Development; PSE, parental self-efficacy.
